# Dimensions of Blue Carbon and emerging perspectives

**DOI:** 10.1098/rsbl.2018.0781

**Published:** 2019-03-06

**Authors:** Catherine E. Lovelock, Carlos M. Duarte

**Affiliations:** 1 School of Biological Sciences, The University of Queensland, St Lucia, Queensland 4072, Australia; 2 King Abdullah University of Science and Technology (KAUST), Red Sea Research Center (RSRC), Thuwal 23955-6900, Saudi Arabia

**Keywords:** mangrove, seagrass, saltmarsh, macroalgae, carbon sequestration, coastal wetlands

## Abstract

*Blue Carbon* is a term coined in 2009 to draw attention to the degradation of marine and coastal ecosystems and the need to conserve and restore them to mitigate climate change and for the other ecosystem services they provide. Blue Carbon has multiple meanings, which we aim to clarify here, which reflect the original descriptions of the concept including (1) all organic matter captured by marine organisms, and (2) how marine ecosystems could be managed to reduce greenhouse gas emissions and thereby contribute to climate change mitigation and conservation. The multifaceted nature of the Blue Carbon concept has led to unprecedented collaboration across disciplines, where scientists, conservationists and policy makers have interacted intensely to advance shared goals. Some coastal ecosystems (mangroves, tidal marshes and seagrass) are established *Blue Carbon ecosystems* as they often have high carbon stocks, support long-term carbon storage, offer the potential to manage greenhouse gas emissions and support other adaptation policies. Some marine ecosystems do not meet key criteria for inclusion within the Blue Carbon framework (e.g. fish, bivalves and coral reefs). Others have gaps in scientific understanding of carbon stocks or greenhouse gas fluxes, or currently there is limited potential for management or accounting for carbon sequestration (macroalgae and phytoplankton), but may be considered Blue Carbon ecosystems in the future, once these gaps are addressed.

## 1. A brief history—the Blue Carbon concept

Research on different processes of the marine carbon cycle was already 100 years old in 1944 [1]. They focused predominantly on the contribution of oceanic phytoplankton, which still prevail in the current depictions of the global carbon cycle [2]. Earlier, however, in 1914 [3] some scientists concluded that seagrass (*Zostera marina*) contributed most of the carbon stocks in Danish coastal sediments, while others drew attention to the role of macrophytes as global carbon sinks [4], and provided a first estimate of their global contribution to carbon storage [5] and burial [6]. Two highly influential reports built on these and other advances in science and policy to describe what they called ‘Blue Carbon’. The volume by Nelleman *et al*. entitled *Blue Carbon. The role of healthy oceans in binding carbon. A rapid response assessment*, gave us a very broad definition of Blue Carbon, starting with the following statement: ‘Out of all the biological carbon (or green carbon) captured in the world, over half (55%) is captured by marine living organisms—not on land—hence it is called Blue Carbon’ [7, p. 6]. The volume by Laffoley & Grimsditch entitled *The management of natural coastal carbon sinks* recognized the role of marine organisms in the capture of CO_2_ within marine ecosystems, but took a pragmatic approach aiming to ‘…quantify the greenhouse gas implications of the management of particular coastal ecosystems, being careful to choose those whose management can be influenced by the application of existing policy agreements and well established area-based management tools and approaches’ [8, p. 1]. Underlying both these reports is the concept that marine ecosystems are important for CO_2_ capture from the atmosphere. Both documents clearly articulated the imperative to focus on conserving and repairing marine ecosystems that contribute to this role, thereby avoiding CO_2_ emissions associated with their destruction and restoring their CO_2_ capture potential, which would also reinstate many important ecosystem services these ecosystems provide.

The multifaceted nature of the Blue Carbon concept has led to a rich, varied and cross-disciplinary research that spans biophysical sciences, conservation, economics, policy and law (see this issue), leading to unprecedented levels of collaboration among contributors in different disciplines, institutions and governments geared toward conserving and restoring coastal ecosystems to mitigate greenhouse gas emissions, promote coastal adaptation to climate change and maintain ecosystem services. However, the multifaceted nature of Blue Carbon has also contributed to confusion and misunderstandings as to what Blue Carbon really is. The study of marine carbon stocks and cycles is important but is only a component of the Blue Carbon concept. The similarity, but divergent emphases of the two early reports [7,8] has propagated over the science and policy landscape as interest in Blue Carbon has grown and there are increasing numbers of contributors with new ideas entering into the discourse.

## 2. Currently actionable Blue Carbon ecosystems

Blue Carbon ecosystems meet a range of criteria (table 1, [9,10]). Mangrove, tidal marsh and seagrass ecosystems align with multiple criteria (table 1, see contributions in this volume). Critical for the development of actionable projects, these ecosystems fall within the IPCC definition of ‘wetlands’ and mangroves are often classified as ‘forests’ (and therefore included in national forest inventories), enabling their inclusion within greenhouse gas accounting guidance of the International Panel on Climate Change (IPCC). The IPCC provided emission factors (CO_2_, methane and nitrous oxide) for land-use change in coastal wetlands for activities that result in loss and conversion or those leading to restoration [11]. Mangroves, where they are included in national forest inventories, may also be included in existing greenhouse gas reduction schemes like Reduced Emissions from Deforestation and Degradation (REDD+) [12]. Carbon markets have developed methodologies that reflect these developments [13–15] and successful projects have been developed [16]. Mangroves, tidal marsh and seagrass are also important ecosystems for climate change adaptation in the coastal zone [17], establishing further compelling reasons for their inclusion as Blue Carbon ecosystems. As a result, after the Paris Agreement (2016), a range of nations have included coastal wetlands in their mitigation activities within their National Determined Contributions [18]. Despite meeting the criteria in table 1, these coastal ecosystems also have a range of characteristics that remain challenges to the development of Blue Carbon projects, including high spatial variation in greenhouse gas emissions, uncertainty around land tenure, tidal boundaries and legislative responsibilities for which research and development are still required [19].

**Table 1.** Assessment of whether coastal ecosystems meet the Blue Carbon criteria (modified from [7,8]). Question marks indicate where additional investigations of the science or policy are needed. Green shading indicates strong evidence for meeting the criteria, yellow indicates some evidence or inference, grey indicates that the criteria are not met. See electronic supplementary material, table S1 for illustrative references (indicated by the superscript numbers) and electronic supplementary material, table S2 for the criteria on which the ecosystems are assessed (either yes, no or inconclusive (?)). A description of the ecosystems listed can be found in the electronic supplementary material, reference 37. GHG, geenhouse gas.

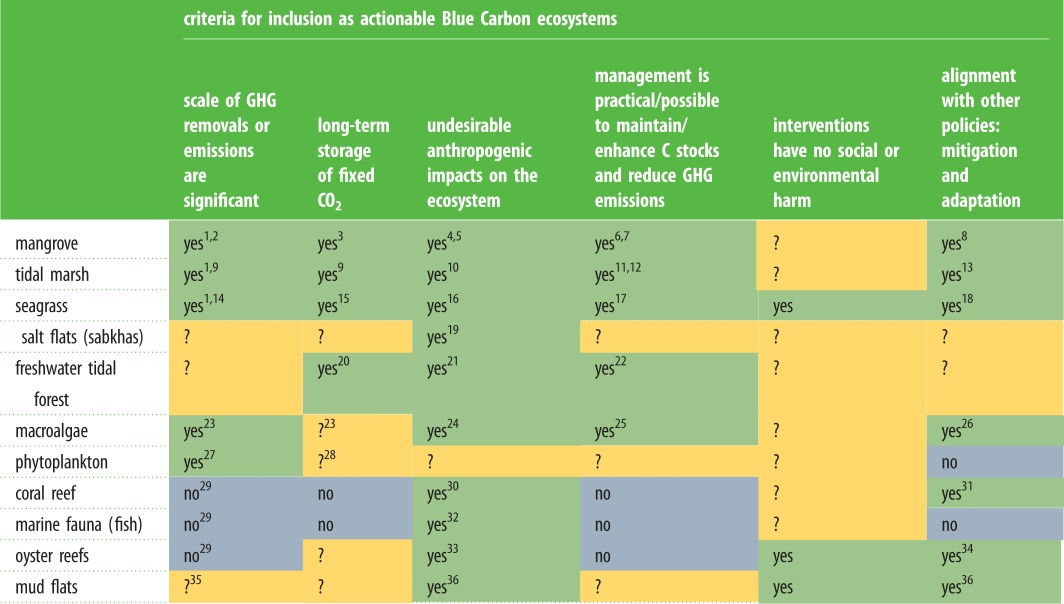

## 3. Ecosystems where Blue Carbon stocks and sequestration rates are being explored (emerging)

**Figure 1.**
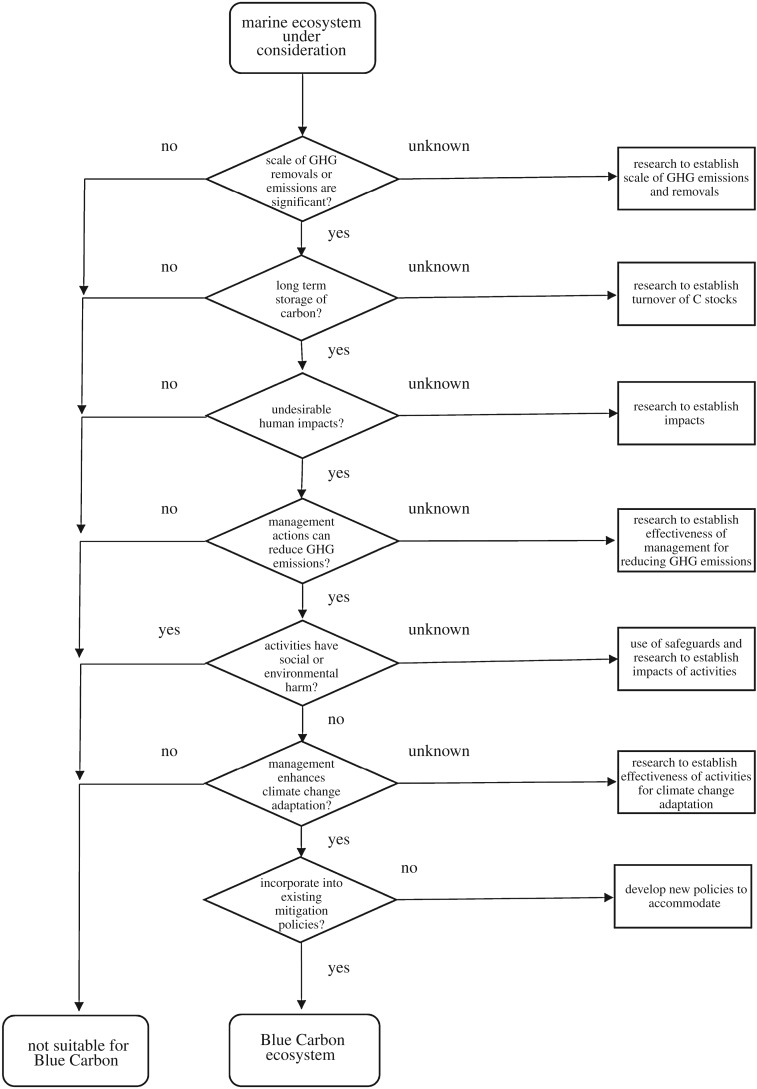
Questions to establish research needs for emerging Blue Carbon ecosystems. GHG, greenhouse gas.

While progress continues in the development of Blue Carbon projects in mangroves, tidal marshes and seagrass, there is debate as to whether other ecosystems, beyond angiosperm-dominated coastal ecosystems, are Blue Carbon ecosystems. Key criteria from table 1 can be used to guide research (figure 1), including whether these habitats can be managed to contribute to climate change mitigation and, if so, can outcomes be achieved through conservation that has adaptation benefits. Habitats dominated by calcifying organisms (e.g. coral reefs, oyster reefs) contribute to climate change adaptation, through energy dissipation and contribution to sediments, but not through greenhouse gas mitigation, as the process of calcification releases CO_2_ and thus these ecosystems are likely to be net CO_2_ sources rather than sinks [20]. However, future research on the role of calcifying organisms in organic matter sequestration could alter this view in the future. Pelagic ecosystems, including those with mobile marine fauna and phytoplankton, have also been suggested to be included as Blue Carbon ecosystems. Their contribution to climate change mitigation through long-term carbon preservation is uncertain and they do not contribute to climate change adaptation. Phytoplankton have been proposed to be used for climate change mitigation since the mid-1990s when the first ocean fertilization experiments were conducted [21]. Fertilizing the ocean with iron to stimulate the production of phytoplankton biomass which then sinks beyond the thermocline to be stored for thousands of years has been controversial mainly because evidence that a large amount of fixed carbon reaches the deep sea is equivocal and because a range of adverse, unintended consequences have been identified [22].

Other coastal ecosystems that are considered as Blue Carbon ecosystems include tidally influenced freshwater forests, for example, bald cypress forests and *Melaleuca* forests, which can have huge soil carbon stocks in their soils and which have been greatly reduced in cover [23]. Sabkhas, which comprise high intertidal salt flats dominated by microbial mats and which can be extensive in arid environments [24], may also be candidates for Blue Carbon-based conservation although information on C stocks and fluxes is currently limited as is information on their role in adaptation to climate change. Kelp and other seaweed beds are also being considered as Blue Carbon ecosystems [25]. There is evidence that seaweeds produce highly recalcitrant compounds [26] and that organic carbon may be buried in sediments or transported to the deep sea and thus stored for thousands of years [25]. In addition to conserving wild kelp beds, seaweed aquaculture offers opportunities for climate change mitigation and adaptation [27]. However, seaweeds and phytoplankton necessitate a different approach to those typically involved in established Blue Carbon ecosystems as they involve management of the fate of the carbon they produce and the locations in which carbon accumulates, which may be distant from the sites of production (e.g. in deep water) [28]. A key step in moving forward to including these ecosystems as Blue Carbon ecosystems is to provide enhanced scientific evidence of carbon storage, how it can be managed and also policy guidance on how the ecosystems may be included in greenhouse gas accounting.

## 4. Conclusion

The Blue Carbon concept is multifaceted, which has been hugely beneficial and facilitated the inclusion of and communication among a wide range of contributors for the benefit of conservation of coastal wetlands. Blue Carbon science has a very broad scope because it seeks to explore all potential opportunities for climate change mitigation and adaption in marine ecosystems. A range of criteria have to be met before some of the proposed Blue Carbon ecosystems can be included in climate change mitigation strategies.

## Supplementary Material

Supplementary Table S1 and S2

## Data accessibility

There are no data associated with this manuscript.
