# Supplementary material for: Dimensions of Blue Carbon and emerging perspectives
Source: Biol Lett. 2019 Mar 6;15(3):20180781. doi: 10.1098/rsbl.2018.0781 (PMC6451379; doi:10.1098/rsbl.2018.0781)
Supplement: Supplementary Table S1 and S2 [file rsbl20180781supp1.docx]

**Supplementary Materials**

Catherine E. Lovelock and Carlos M. Duarte. Dimensions of Blue Carbon and emerging perspectives

Illustrative references (Table S2) and criteria on which the ecosystems are assessed (either YES, NO, or inconclusive?) (Table S2).

Table S1. References supporting Table 1 and Table S2.

| 1 | Pendleton L, Donato DC, Murray BC, et al. 2012. Estimating Global “Blue Carbon” emissions from conversion and degradation of vegetated coastal ecosystems. PLoS One 7(9): e43542 |
| --- | --- |
| 2 | Murdiyarso, D., Purbopuspito, J., Kauffman, J. B., Warren, M. W., Sasmito, S. D., Donato, D. C., ... & Kurnianto, S. (2015). The potential of Indonesian mangrove forests for global climate change mitigation. Nature Climate Change, 5(12), 1089. |
| 3 | McKee, K. L., Cahoon, D. R., & Feller, I. C. (2007). Caribbean mangroves adjust to rising sea level through biotic controls on change in soil elevation. Global Ecology and Biogeography, 16(5), 545-556. |
| 4 | Atwood, T. B., Connolly, R. M., Almahasheer, H., Carnell, P. E., Duarte, C. M., Lewis, C. J. E., ... & Serrano, O. (2017). Global patterns in mangrove soil carbon stocks and losses. Nature Climate Change, 7(7), 523. |
| 5 | Donato DC, Kauffman JB, Murdiyarso D, et al. 2011. Mangroves among the most carbon-rich tropical forests and key in land-use carbon emissions. Nat Geosci 4:293-297. |
| 6 | Kauffman JB, Heider C, Norfolk J, and Payton F. 2014. Carbon stocks of intact mangroves and carbon emissions arising from their conversion in the Dominican Republic. Ecol Applic 24: 518-527. |
| 7 | Osland, M. J., Spivak, A. C., Nestlerode, J. A., Lessmann, J. M., Almario, A. E., Heitmuller, P. T., ... & Harvey, J. E. (2012). Ecosystem development after mangrove wetland creation: plant–soil change across a 20-year chronosequence. Ecosystems, 15(5), 848-866. |
| 8 | Sutton-Greir AE, Moore AK, Wiley PC, et al. 2014. Incorporating ecosystem services into the implementation of existing US natural resource management regulations: Operationalizing carbon sequestration and storage. Mar Pol 43: 246-253. |
| 9 | Macreadie, P. I., Ollivier, Q. R., Kelleway, J. J., Serrano, O., Carnell, P. E., Lewis, C. E., ... & Duarte, C. M. (2017). Carbon sequestration by Australian tidal marshes. Scientific reports, 7, 44071. |
| 10 | Gedan KB, Silliman BR, and Bertness MD. 2009. Centuries of human-driven change in salt marsh ecosystems. Ann Rev Mar Sci 1:117-141. |
| 11 | Mcleod, E., Chmura, G. L., Bouillon, S., Salm, R., Björk, M., Duarte, C. M., Lovelock C.E.. & Silliman, B. R. (2011). A blueprint for Blue Carbon: toward an improved understanding of the role of vegetated coastal habitats in sequestering CO_2_. Frontiers in Ecology and the Environment, 9(10), 552-560. |
| 12 | Kroeger, K. D., Crooks, S., Moseman-Valtierra, S., & Tang, J. (2017). Restoring tides to reduce methane emissions in impounded wetlands: A new and potent Blue Carbon climate change intervention. *Scientific Reports*, *7*(1), 11914. |
| 13 | Shepard, C. C., Crain, C. M., & Beck, M. W. (2011). The protective role of coastal marshes: a systematic review and meta-analysis. PloS one, 6(11), e27374. |
| 14 | Fourqurean JW, Duarte CM, Kennedy H, et al. 2012. Seagrass ecosystems as a globally significant carbon stock. Nat Geosci 5:505-509 |
| 15 | Mateo, M. A., Romero, J., Pérez, M., Littler, M. M., & Littler, D. S. (1997). Dynamics of millenary organic deposits resulting from the growth of the Mediterranean seagrass *Posidonia oceanica*. Estuarine, Coastal and Shelf Science, 44(1), 103-110. |
| 16 | Waycott M, Duarte CM, Carruthers TJB, et al. 2009. Accelerating loss of seagrasses across the globe threatens coastal ecosystems. Proc Nat Acad Sci USA 106:12377-12381 |
| 17 | Marbà N, Arias-Ortiz A, Masqué P, et al. 2015. Impact of seagrass loss and subsequent revegetation on carbon sequestration and stocks. J Ecol 103: 296-302. |
| 18 | Duarte CM, Losada IJ, Hendriks IE, Mazarrasa I. & Marbà. N. 2013. The role of coastal plant communities for climate change mitigation and adaptation. Nat Clim Change 3:961-968. |
| 19 | Lokier, S. W. (2013). Coastal sabkha preservation in the Arabian Gulf. *Geoheritage*, *5*(1), 11-22. |
| 20 | Krauss, K. W., Noe, G. B., Duberstein, J. A., Conner, W. H., Stagg, C. L., Cormier, N., ... & Doyle, T. W. (2018). The Role of the Upper Tidal Estuary in Wetland Blue Carbon Storage and Flux. Global Biogeochemical Cycles. |
| 21 | Bowman, D. M., Prior, L. D., & De Little, S. C. (2010). Retreating Melaleuca swamp forests in Kakadu National Park: Evidence of synergistic effects of climate change and past feral buffalo impacts. *Austral Ecology*, *35*(8), 898-905. |
| 22 | Tran, D. B., Dargusch, P., Herbohn, J., & Moss, P. (2013). Interventions to better manage the carbon stocks in Australian *Melaleuca* forests. *Land Use Policy*, *35*, 417-420. |
| 23 | Krause-Jensen, D., and Carlos M. Duarte. 2016. Substantial role of macroalgae in marine carbon sequestration. Nature Geoscience 9, 737–742, DOI: 10.1038/NGEO2790 |
| 24 | Connell, S. D., Russell, B. D., Turner, D. J., Shepherd, S. A., Kildea, T., Miller, D., ... & Cheshire, A. (2008). Recovering a lost baseline: missing kelp forests from a metropolitan coast. *Marine Ecology Progress Series*, *360*, 63-72. |
| 25 | Wilson, K. C., & North, W. J. (1983). A review of kelp bed management in southern California. *Journal of the World Aquaculture Society*, *14*(1‐4), 345-359. |
| 26 | Koch, E. W., Barbier, E. B., Silliman, B. R., Reed, D. J., Perillo, G. M., Hacker, S. D., ... & Halpern, B. S. (2009). Non‐linearity in ecosystem services: temporal and spatial variability in coastal protection. *Frontiers in Ecology and the Environment*, *7*(1), 29-37. |
| 27 | Falkowski, P., Scholes, R. J., Boyle, E. E. A., Canadell, J., Canfield, D., Elser, J., ... & Mackenzie, F. T. (2000). The global carbon cycle: a test of our knowledge of earth as a system. *Science*, *290*(5490), 291-296. |
| 28 | Durkin, C. A., Van Mooy, B. A., Dyhrman, S. T., & Buesseler, K. O. (2016). Sinking phytoplankton associated with carbon flux in the Atlantic Ocean. *Limnology and Oceanography*, *61*(4), 1172-1187. |
| 29 | Macreadie, P. I., Serrano, O., Maher, D. T., Duarte, C. M., & Beardall, J. (2017). Addressing calcium carbonate cycling in Blue Carbon accounting. Limnology and Oceanography Letters, 2(6), 195-201. |
| 30 | Hughes, T. P., Barnes, M. L., Bellwood, D. R., Cinner, J. E., Cumming, G. S., Jackson, J. B., ... & Palumbi, S. R. (2017). Coral reefs in the Anthropocene. *Nature*, *546*(7656), 82. |
| 31 | Ferrario, F., Beck, M. W., Storlazzi, C. D., Micheli, F., Shepard, C. C., & Airoldi, L. (2014). The effectiveness of coral reefs for coastal hazard risk reduction and adaptation. *Nature Communications*, *5*, 3794. |
| 32 | Pauly, D., & Zeller, D. (2016). Catch reconstructions reveal that global marine fisheries catches are higher than reported and declining. *Nature Communications*, *7*, ncomms10244. |
| 33 | Beck, M. W., Brumbaugh, R. D., Airoldi, L., Carranza, A., Coen, L. D., Crawford, C., ... & Lenihan, H. S. (2011). Oyster reefs at risk and recommendations for conservation, restoration, and management. *Bioscience*, *61*(2), 107-116. |
| 34 | Piazza, B. P., Banks, P. D., & La Peyre, M. K. (2005). The potential for created oyster shell reefs as a sustainable shoreline protection strategy in Louisiana. *Restoration Ecology*, *13*(3), 499-506. |
| 35 | Bridgham, S. D., Megonigal, J. P., Keller, J. K., Bliss, N. B., & Trettin, C. (2006). The carbon balance of North American wetlands. Wetlands, 26(4), 889-916. |
| 36 | Murray, N.J., Phinn, S.R., DeWitt, M., Ferrari, R., Johnston, R., Lyons, M.B., Clinton, N., Thau, D. and Fuller, R.A. (2018). The global distribution and trajectory of tidal flats. Nature, <https://doi.org/10.1038/s41586-018-0805-8> |
| 37 | Millennium Ecosystem Assessment, 2005. Ecosystems and human well-being: Wetlands and water Synthesis. World Resources Institute, Washington, DC. |
| 38 | Crooks, S., Emmer, I., Murdiyarso, D., & Brown, B. (2014). Guiding principles for delivering coastal wetland carbon projects. Report to the United Nations Environment Program and the Center for International Forestry Research. |
| 39 | Fletcher, R., Dressler, W., Büscher, B., & Anderson, Z. R. (2016). Questioning REDD+ and the future of market‐based conservation. *Conservation Biology*, 30(3), 673-675. |

Table S2 Criteria used for assessing whether ecosystems are considered Blue Carbon ecosystems.

| Criteria | Key considerations |
| --- | --- |
| Scale of GHG gas removals or emissions are significant | Multiple sources of scientific evidence is available and supported by theory, that the ecosystem have either 1) high levels of carbon (C) stocks either globally or locally (on an area basis); 2) high rates of carbon sequestration globally or locally. E.g. we conclude “YES” for mangroves which have a limited global cover but high levels of soil C stocks on an aerial basis, and also for phytoplankton which have extensive global cover but low C stocks on an area basis. |
| Long term storage of fixed CO_2_ | Multiple sources of scientific evidence that fixed organic carbon is stored for longer than 100 years. |
| Undesirable anthropogenic impacts on the ecosystem | Multiple sources of evidence that ecosystems have been removed, converted or degraded by human activities. E.g. we conclude “YES” for seagrass which have reduced cover caused by declining coastal water quality; but “?” for phytoplankton, because cover has not been reduced, but there is evidence that phytoplankton communities have changed in coastal oceans due to nutrient enrichment, yet we are uncertain of the spatial extent or functional consequences of this change. |
| Management is practical/ possible to maintain/enhance C stocks and reduce GHG emissions | Multiple sources of evidence that ecosystems can be managed to either maintain or enhance carbon stocks or to decrease net greenhouse gas emissions. E.g. we conclude “YES” for tidal marshes that can be restored, thereby protecting remaining soil C stocks, enhancing soil C sequestration and, if there were significant methane emissions from freshwater ecosystems prior to restoration, also potentially through a net reduction in methane emissions; but “?” for phytoplankton where there is conflicting evidence on the levels of C sequestration can be achieved through fertilizer additions; and “NO” for ecosystems where high levels of calcification, which result in CO_2_ emissions, and where we assume management is focussed on enhancing growth of habitat forming calcifying organisms. |
| Interventions have no social or environmental harm | Blue Carbon projects should follow the recommendations of Crooks et al. (2014)[38] thereby avoiding the negative social consequences that have been associated with programs such as REDD+[39]. However, risks of social harm and/or environmental harm with their restoration and/or conservation cannot be completely eliminated. Therefore, to reflect these uncertainties, and based on the restoration literature, as well as literature on marine protected areas, we assign a “?” to for mangroves, tidal marshes, coral reefs and marine fauna. We assign “YES” to seagrass, oyster reefs and mud flats as we are not aware of reports of adverse social or environmental harm associated with their conservation or restoration. |
| Alignment with other policies: mitigation and adaptation | Blue Carbon project outcomes align with international conventions, national or sub-national policies and guidelines. E.g. Sustainable Development Goals, Convention on Biodiversity, RAMSAR Convention, climate adaptation policies, local biodiversity conservation, fishery habitat protection and others. |
